# Progressive implementation of patient blood management and declining transfusion rates in major orthopedic surgery. A single-centre experience

**DOI:** 10.3389/fsurg.2026.1918812

**Published:** 2026-10-02

**Authors:** Matic Kolar, Boštjan Kocjančič

**Affiliations:** 1 Department of Orthopaedic Surgery, University Medical Centre Ljubljana, Ljubljana, Slovenia; 2 Faculty of Medicine, University of Ljubljana, Ljubljana, Slovenia

**Keywords:** arthroplasty, orthopaedic surgery, patient blood management, spine, tranexamic acid, transfusion

## Abstract

**Introduction:**

Patient Blood Management (PBM) has become an essential component of contemporary perioperative medicine. Major orthopedic surgery has historically been associated with substantial perioperative blood loss and high allogeneic transfusion rates. The aim of this study was to describe the temporal association between the progressive implementation of a multidisciplinary PBM programme and transfusion rates in major orthopedic surgery at our institution.

**Materials and methods:**

This retrospective observational single-centre study on annual institutional aggregated data included patients undergoing major orthopedic procedures, including total hip arthroplasty (THA), total knee arthroplasty (TKA), and major spine surgery (MSS), between 2012 and 2018. The PBM programme incorporated a multidisciplinary perioperative approach consisting of preoperative anemia management, restrictive transfusion thresholds, a single-unit transfusion policy, routine tranexamic acid (TXA) administration, meticulous surgical hemostasis, minimally invasive surgical techniques, and selective use of intraoperative cell salvage systems. Annual transfusion rates and PBM-related interventions were analysed over the study period for the overall cohort and separately for each procedure group.

**Results:**

A total of 7,439 major orthopedic procedures were analysed. Overall transfusion rates decreased from 38.1% in 2012 to 9.2% in 2018, corresponding to an absolute reduction of 28.9% and a relative reduction of 76.0%. Similar reductions were observed for THA (45.0% to 13.6%), TKA (31.7% to 4.6%), and MSS (32.1% to 9.1%). Logistic regression demonstrated that each additional calendar year was associated with a significant reduction in the odds of transfusion in the overall cohort (OR 0.72, 95% CI 0.70–0.74; *p* < 0.001), with comparable trends observed for each surgical subgroup. During the same period, TXA utilization increased progressively, reaching a mean of 2.1 g per procedure in 2018, whereas intraoperative cell saver use became increasingly selective.

**Conclusion:**

The present study demonstrates that multidisciplinary PBM implementation substantially reduces transfusion requirements in major orthopedic surgery. PBM should not be regarded as a single intervention, but rather as a coordinated perioperative strategy based on synergistic implementation of multiple blood conservation measures, resulting in improved perioperative management.

## Introduction

Patient Blood Management (PBM) is defined as an evidence-based multidisciplinary, multimodal and patient-tailored approach designed to optimize treatment outcomes through preservation and management of a patient's own blood resources, while minimising the need for allogeneic transfusion (1). The term PBM was first used in 2005 by Isbister, who realized the importance to change the focus of transfusion medicine from blood products to the patients (2). Subsequently, PBM became an integral component of perioperative medicine, with its principles being progressively implemented into clinical practice and increasingly supported by subspecialists involved in perioperative patient care, including but not limited to general practitioners, anesthesiologists, transfusion medicine specialists, and surgeons (3).

Major orthopedic surgery has historically been associated with substantial perioperative blood loss and relatively high transfusion rates exceeding 30% following primary and revision total joint arthroplasty (TJA) (4). Preoperative anemia, reported in approximately 25%–35% of patients undergoing major orthopedic procedures, represents an independent predictor of increased postoperative morbidity, mortality, delayed functional recovery, prolonged hospitalization, and higher risk of perioperative transfusion. Reduced hemoglobin concentration compromises tissue oxygen delivery and may adversely affect postoperative recovery and clinical outcomes (5–7). Although allogeneic blood transfusion may be necessary in selected clinical situations, its perioperative use has consistently been associated with increased morbidity, infectious complications, prolonged hospital stay, and higher healthcare-related costs (6, 7). In addition to well-recognized risks such as acute transfusion reactions, transfusion-related acute lung injury, transfusion-associated circulatory overload, immunomodulation, and transmission of infectious agents, allogeneic transfusion has also been linked to higher rates of postoperative infection and poorer overall clinical outcomes following major orthopedic procedures (6–9). Furthermore, transfused patients have been shown to incur significantly greater treatment expenditures compared with non-transfused patients undergoing similar surgery (9). These findings have contributed to the increasing implementation of PBM strategies.

Contemporary PBM programmes are founded on three principal strategies: optimization of erythropoiesis and treatment of preoperative anemia, reduction of perioperative blood loss, and enhancement of the patient's physiological tolerance to anemia (10). Within orthopedic surgery, PBM measures primarily focus on meticulous surgical hemostasis, minimally invasive surgical approaches, standardized transfusion algorithms, restrictive transfusion thresholds, and pharmacological blood conservation techniques aimed at reducing unnecessary exposure to allogeneic blood products (11). Among these interventions, a synthetic antifibrinolytic agent TXA, has become one of the most important and widely adopted components of contemporary orthopedic PBM protocols. A substantial body of evidence, including multiple randomized controlled trials and large meta-analyses, has demonstrated that intravenous, topical, and combined TXA administration significantly reduces perioperative blood loss, postoperative hemoglobin decline, and allogeneic transfusion requirements in both THA and TKA without increasing the risk of thromboembolic complications. Owing to its efficacy, favorable safety profile, and cost-effectiveness, TXA is currently regarded as a standard component of perioperative blood conservation strategies in major orthopedic surgery (12–14).

The aim of this study was to describe the long-term institutional trends in allogeneic transfusion rates accompanying the progressive implementation of a multidisciplinary PBM programme in a university tertiary orthopedic centre between 2012 and 2018, and to report the individual components of the programme.

## Materials and methods

A retrospective observational study was performed at the department of orthopedic surgery in a main national tertiary university medical centre. Institutional data from January 2012 until December 2018 were retrospectively reviewed. The unit of analysis was the calendar-year institutional aggregate.

Patients undergoing THA, TKA, and MSS were included in the analysis. Revision arthroplasty and emergency procedures were excluded. The PBM program was progressively implemented during the study period and included several coordinated interventions: preoperative screening and treatment of anemia and iron deficiency, collaboration with general practitioners and anesthesiologists, routine use of TXA (intravenous and intra-articular applications) and local infiltration analgesia (LIA), neuraxial anesthesia, meticulous intraoperative hemostasis, minimally invasive surgical techniques, restrictive transfusion thresholds, single-unit transfusion policy, selective use of intraoperative cell saver systems. Transfusion decisions progressively shifted from liberal transfusion practices toward symptom-based restrictive transfusion criteria according to modern PBM recommendations.

Its components were as follows.

Preoperative anemia management. Patients were assessed at the preoperative anesthesia clinic, and those found to be anemic were referred to their general practitioner for evaluation and treatment before elective surgery, with optimization of hemoglobin towards a target concentration of 130 g/L. Iron deficiency was corrected with iron supplementation, whereas erythropoiesis-stimulating agents were used only exceptionally.
Pharmacological blood conservation. TXA was administered intravenously and intra-articularly, in accordance with the combined clinical practice guidelines on TXA use in total joint arthroplasty (15). One unit corresponded to one ampoule containing 500 mg. TXA was first used in 2014, its use widened progressively over the following years, and from 2016 onwards it was administered in essentially all eligible procedures. Patients with a history of thromboembolic disease or other recognized contraindications did not receive TXA.
Anesthetic measures. Neuraxial anesthesia was preferred whenever feasible, and LIA containing a local anesthetic combined with epinephrine was used routinely in TJA.
Surgical measures. These comprised minimally invasive approaches where feasible, minimization of soft-tissue trauma, early control of bleeding and meticulous hemostasis, attention to patient positioning, maintenance of normothermia and control of arterial blood pressure, and a progressive reduction in the use of postoperative wound drains.
Transfusion strategy. The transfusion threshold was progressively lowered from the historical hemoglobin concentration of 100 g/L, derived from the 10/30 rule, towards 80 g/L, with individualized, symptom-based decisions (16, 17). A higher threshold was maintained in patients with cardiac disease or with clinical signs of poor tolerance of anemia, including tachycardia, hypotension, dyspnea, new-onset arrhythmia or angina, whereas lower concentrations were accepted in young, hemodynamically stable patients. A single-unit policy was applied, in which one unit was ordered and administered at a time and the patient reassessed clinically and by hemoglobin measurement before any further unit was given; transfusion decisions were based on the patient's symptoms rather than on the hemoglobin concentration alone.
Autologous techniques. Intraoperative cell salvage was used selectively and was mainly reserved for procedures with anticipated blood loss of approximately 1,000 mL or more.

### Statistical analysis

Annual institutional aggregated data regarding the number of procedures, allogeneic transfusions, TXA utilization, and intraoperative cell saver use were collected for each calendar year. Transfusion rates were calculated for the overall cohort and separately for patients undergoing THA, TKA, and MSS. Descriptive statistics were used to summarize annual institutional activity and PBM-related interventions. Temporal trends in transfusion rates were assessed using the Cochran–Armitage trend test. In addition, grouped binomial logistic regression was performed with calendar year entered as a continuous predictor to evaluate the annual change in the odds of transfusion. Odds ratios (OR) with 95% confidence intervals (CI) were calculated for each additional calendar year. Absolute (ARR) and relative (RRR) risk reductions in transfusion rates between 2012 and 2018 were also determined. Confidence intervals for annual transfusion proportions were calculated using the normal approximation to the binomial distribution. All analyses were unadjusted, as no patient-level covariates were available; the results therefore describe temporal trends in institutional transfusion practice rather than adjusted treatment effects. Statistical analyses were performed using SPSS ver. 25 (IBM, Armonk, NY, USA). A *p* value < 0.05 was considered statistically significant.

## Results

A total of 7,439 major orthopedic procedures were performed during the study period, including 3,382 THA, 2,668 TKA, and 1,389 MSS. Overall, 1,563 patients (21.0%) received at least one allogeneic blood transfusion during the study period. Over the entire period, allogeneic transfusion was administered in 844 THA procedures (25.0%), 435 TKA procedures (16.3%) and 284 MSS procedures (20.4%) (Table 1).

**Table 1:** Annual number of procedures and transfusion rates according to procedure type.

Year	THA, n	THA transfused, *n* (%)	TKA, n	TKA transfused, *n* (%)	MSS, n	MSS transfused, *n* (%)
2012	509	229 (45.0)	363	115 (31.7)	193	62 (32.1)
2013	486	192 (39.5)	379	109 (28.8)	210	64 (30.5)
2014	487	139 (28.5)	369	83 (22.5)	214	54 (25.2)
2015	450	77 (17.1)	340	40 (11.8)	197	28 (14.2)
2016	442	61 (13.8)	340	32 (9.4)	178	34 (19.1)
2017	558	85 (15.2)	444	36 (8.1)	211	25 (11.8)
2018	450	61 (13.6)	433	20 (4.6)	186	17 (9.1)
Total	3,382	844 (25.0)	2,668	435 (16.3)	1,389	284 (20.4)

Overall transfusion rates decreased from 38.1% (406/1,065) in 2012 to 9.2% (98/1,069) in 2018, corresponding to an absolute reduction of 28.9% and a relative reduction of 76.0%. A significant downward temporal trend was observed with Cochran–Armitage trend test (*p* < 0.001). Logistic regression demonstrated that each additional calendar year was associated with a 28% reduction in the odds of transfusion (OR 0.72, 95% CI 0.70–0.74; *p* < 0.001) (Table 2).

**Table 2:** Magnitude of transfusion reduction according to procedure type.

Procedure	2012	2018	ARR	RRR	OR/year (95% CI)	p
Overall	38.1	9.2	28.9	76.0	0.72 (0.70–0.74)	<0.001
THA	45.0	13.6	31.4	69.9	0.73 (0.69–0.76)	<0.001
TKA	31.7	4.6	27.1	85.4	0.68 (0.65–0.72)	<0.001
MSS	32.1	9.1	23.0	71.5	0.77 (0.72–0.83)	<0.001

Similar reductions were observed across all surgical subgroups. In patients undergoing THA, transfusion rates decreased from 45.0% (229/509) to 13.6% (61/450), representing an absolute reduction of 31.4 percentage points and a relative reduction of 69.9% (OR 0.73, 95% CI 0.69–0.76; *p* < 0.001). In TKA, transfusion rates declined from 31.7% (115/363) to 4.6% (20/433), corresponding to the largest relative reduction (85.4%) and a 32% annual reduction in transfusion odds (OR 0.68, 95% CI 0.65–0.72; *p* < 0.001). In MSS, transfusion rates decreased from 32.1% (62/193) to 9.1% (17/186), representing a relative reduction of 71.5% (OR 0.77, 95% CI 0.72–0.83; *p* < 0.001) (Table 2).

Simultaneously, TXA administration increased substantially. One unit corresponded to one ampoule containing 500 mg of TXA. Institutional consumption increased from none in 2012 to 4,536 units in 2018, corresponding to a mean of 4.2 units, or 2.1 g, per procedure, which is consistent with routine combined intravenous and intra-articular administration in essentially all eligible patients by the end of the study period. Cell saver use became increasingly selective and was reserved mainly for procedures with anticipated major blood loss. Its utilization dropped from 16.3% to 5.6% of cases. In Table 3, annual transfusion rates are presented together with the proportion of procedures in which TXA and intraoperative cell saver systems were used. The temporal trends in transfusion rates for the overall cohort and individual surgical subgroups are illustrated in Figure 1.

**Table 3:** Annual transfusion rates and utilization of TXA and cell saver systems in major orthopedic surgical procedures.

Year	No. surgeries	No. transfusion (%)	95% CI	No. cell saver (%)	No. TXA units	Mean TXA dose per procedure (g)
2012	1,065	406 (38.1)	35.2–41.1	174 (16.3)	0	**0**
2013	1,075	365 (34.0)	31.2–36.8	126 (11.7)	**10**	**<0**.**01**
2014	1,070	276 (25.8)	23.2–28.5	78 (7.3)	**760**	**0**.**36**
2015	987	145 (14.7)	12.5–17.0	36 (3.6)	1,200	**0**.**61**
2016	960	127 (13.2)	11.1–15.5	72 (7.5)	3,713	**1**.**93**
2017	1,213	146 (12.0)	10.3–14.0	72 (5.9)	4,627	**1**.**91**
2018	1,069	98 (9.2)	7.6–11.1	60 (5.6)	4,536	**2**.**12**

One TXA unit corresponds to 500 mg.

**Figure 1:**
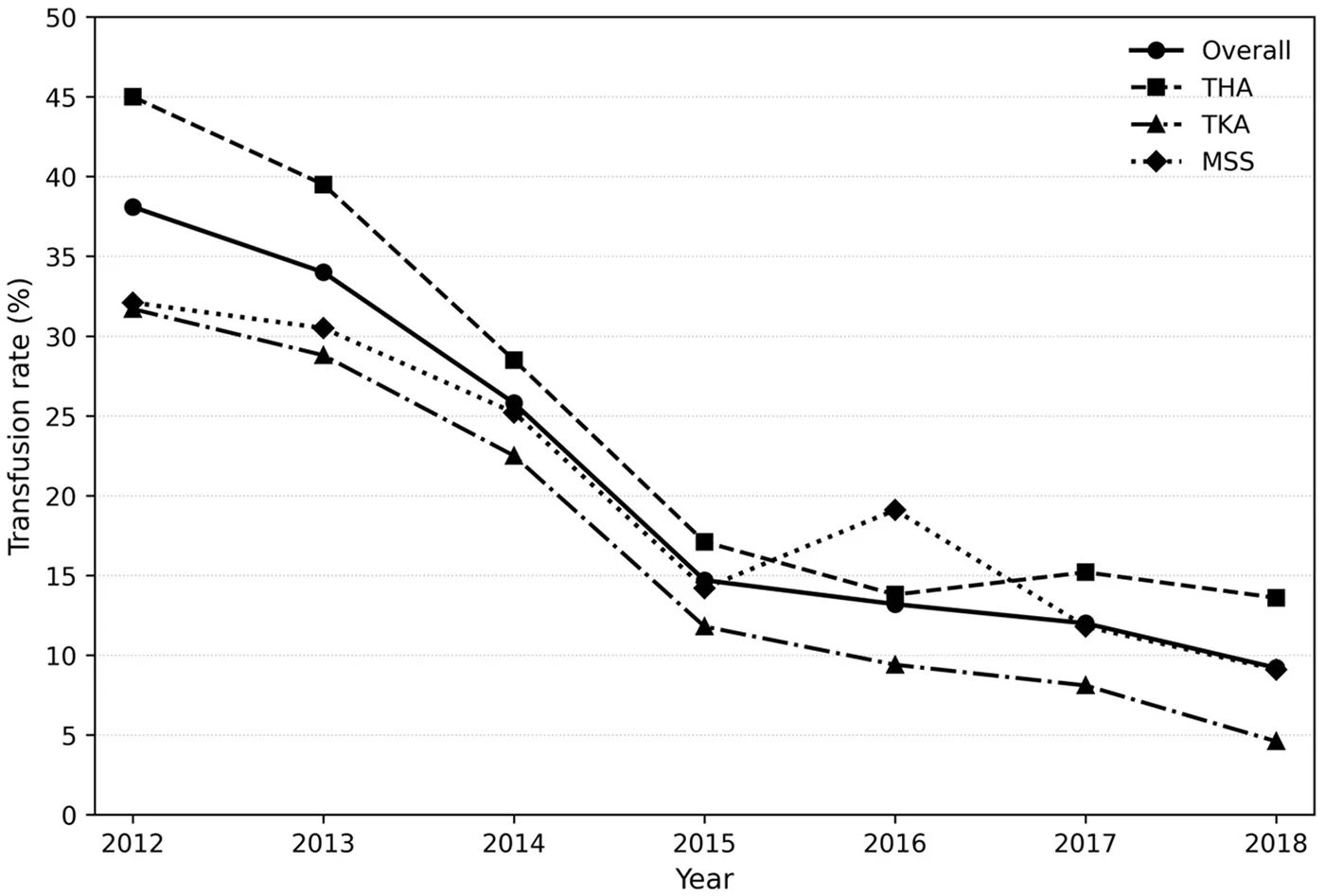
Temporal trends in transfusion rates for the overall cohort and individual surgical subgroups.

## Discussion

The present study documents a substantial, progressive and statistically consistent decline in allogeneic transfusion rates in major orthopedic surgery, which paralleled the stepwise implementation of a multidisciplinary PBM programme. Beyond the substantial absolute and relative reductions in transfusion rates, temporal trend analyses further demonstrated the consistency of this decline throughout the study period. Logistic regression showed that each additional calendar year was associated with a 28% reduction in the odds of allogeneic transfusion in the overall cohort. Similar significant trends were observed across all surgical subgroups, with annual reductions in transfusion odds of 32% for TKA, 27% for THA, and 23% for MSS. These findings suggest that the reduction in transfusion requirements was not confined to isolated changes between the beginning and end of the study period but reflected a sustained and progressive improvement over time with gradual implementation of PBM strategies.

Several perioperative interventions have likely contributed synergistically to the substantial reduction in transfusion rates observed both in our cohort and in contemporary orthopedic practice. Recent studies based on large multi-institutional databases have reported allogeneic transfusion rates of approximately 9% following THA and 4% following TKA (16). Following the implementation of structured PBM strategies, transfusion rates in our institution approached these contemporary benchmarks, decreasing to 13.6% for THA and 4.6% for TKA. Importantly, prior to PBM implementation, transfusion rates in our cohort were substantially higher, particularly in THA patients, where transfusions were administered in 45%. Therefore, despite still slightly exceeding averages in the THA population, implementation of those strategies resulted in a marked relative reduction in transfusion requirements, reaching approximately 69% in THA and as much as 85% in TKA patients. The persistently somewhat higher transfusion rate observed in the THA cohort likely reflects the specific characteristics of our institution as the principal national tertiary referral and university centre, where a substantial proportion of patients present with significant comorbidities and elevated perioperative risk profiles. Consequently, our centre manages a disproportionate number of complex and polymorbid patients, including individuals considered too high-risk for primary arthroplasty procedures at smaller regional hospitals.

One of the most important advances within PBM has been the increased recognition, evaluation, and treatment of preoperative anemia. It has consistently been identified as an independent risk factor for perioperative transfusion, postoperative complications, prolonged hospitalization, delayed recovery, and increased mortality (5–7). Consequently, optimization of hemoglobin levels before surgery has become essential part of perioperative care, aiming to improve physiological reserve and reduce exposure to allogeneic blood products. Structured quality-improvement programmes built on the same principle have previously been shown to reduce both preoperative anemia and transfusion requirements after primary hip and knee arthroplasty (18, 19).

Another major shift has occurred in transfusion practice itself. Historically, transfusion strategies were mainly guided by the “10/30 rule” introduced by Adams and Lundy in 1942, recommending transfusion at a hemoglobin threshold of 10 g/dL or hematocrit of 30% (17). Although widely accepted for decades, accumulating evidence over the last two decades has shown that restrictive transfusion strategies are safe and effective in hemodynamically stable patients and are associated with lower transfusion rates without compromising clinical outcomes. Our institutional practice followed this evolution, with the transfusion threshold decreasing from 100 g/L towards 80 g/L, individualized symptom-based decision-making, and higher thresholds retained in patients with cardiac disease. This is consistent with the target haemoglobin range of 7–9 g/dL recommended by the European Society of Anaesthesiology during active bleeding, and in line with the recommended threshold of 8 g/dL for patients undergoing orthopedic surgery. In parallel, implementation of single-unit transfusion policies has further promoted more rational and individualized blood utilization by encouraging clinical reassessment after each administered unit (18–22). Pharmacological blood conservation has also become a cornerstone of PBM, particularly through the widespread adoption of TXA. Multiple randomized studies and meta-analyses have confirmed that TXA significantly reduces perioperative blood loss and allogeneic transfusion requirements in TJA without increasing the incidence of thromboembolic complications (12–14). The combined regimen of intravenous and intra-articular administration used at our institution, corresponding to approximately 2 g per procedure by the end of the study period, is in accordance with the clinical practice guidelines on TXA in TJA (15).

In addition to anemia management and restrictive transfusion protocols, continuous advances in surgical technique have further optimized perioperative blood preservation. Minimally invasive approaches, improved electrocautery devices, meticulous surgical hemostasis, and tissue-preserving operative strategies have collectively contributed to reduced operative trauma and lower blood loss. Furthermore, there is accumulating evidence that local infiltration analgesia (LIA) may also support PBM strategies beyond its established role in postoperative pain control and early mobilization. Recent studies have demonstrated that periarticular infiltration protocols containing local anesthetics combined with epinephrine potentially improve local hemostasis during TJA procedures. Preoperative administration of LIA has been associated with a significant reduction in perioperative blood loss during TKA, while combined TXA and LIA protocols have demonstrated reductions in postoperative hemoglobin decline, shorter hospital stay, and lower transfusion rates (23, 24).

Several considerations argue for caution before attributing the observed decline to the PBM programme alone. The study was retrospective and observational, no concurrent control group was available, and the analysis could not be adjusted for changes in patient characteristics or surgical complexity. Transfusion rates in TJA declined substantially during the same period in large multi-institutional databases, irrespective of formally structured PBM programmes, reflecting broader changes in perioperative practice, including shorter operative times, wider use of neuraxial anesthesia, enhanced recovery pathways, earlier mobilization, reduced use of postoperative drains, and the continuing evolution of implants and instrumentation. Moreover, the individual components of the programme were introduced progressively and concurrently, so that their separate contributions cannot be disentangled; the temporal coincidence between the steepest fall in transfusion rates and the first year of substantial TXA use is suggestive but does not establish causality. The observed reduction should therefore be interpreted as a temporal association within a real-world institutional cohort rather than as a demonstrated treatment effect.

The economic impact of PBM is also considerable. While allogeneic transfusion is associated with substantial direct and indirect healthcare costs, including blood processing, crossmatching, administration, prolonged hospitalization, and management of complications, TXA represents a highly cost-effective intervention. At our institution the acquisition cost of one unit of allogeneic red blood cells is approximately €110, with a further €25 for reservation and crossmatching, compared with €0.86 per 500 mg ampoule of TXA or €100 per intraoperative cell salvage set. The TXA administered per procedure at the end of the study period therefore cost approximately €3.6, that is less than one twenty-fifth of the acquisition cost of a single unit of red blood cells, before the indirect costs of transfusion are taken into account. These figures refer to acquisition costs at our institution and are presented as an illustrative comparison; a formal cost-effectiveness analysis, which would require patient-level resource use and outcome data, was beyond the scope of the present study.

The present analysis did not include clinical or safety outcomes. Postoperative hemoglobin concentrations, thromboembolic and infectious complications, length of hospital stay, readmission and mortality were not part of the aggregated dataset. This is of particular relevance given the marked increase in TXA exposure over the study period. Published randomized trials and large observational analyses, have not demonstrated an increase in thromboembolic complications associated with perioperative TXA administration (12–14). Our data can neither confirm nor refute these findings at institutional level, and a prospective patient-level audit including venous thromboembolism, periprosthetic joint infection and length of hospital stay is planned as the next step of our institutional PBM programme.

This study has several limitations. First, its retrospective single-centre design limits the generalizability of the findings. Second, the present study represents a retrospective longitudinal institutional analysis based on annual aggregated institutional data rather than individual patient-level records. Consequently, detailed demographic and clinical characteristics, including age, sex, body mass index, American Society of Anesthesiologists (ASA) classification, preoperative haemoglobin levels, comorbidities, and other patient-specific risk factors, were not consistently available throughout the study period and could therefore not be incorporated into the analyses. Adjustment for potential confounding variables was therefore not feasible, and the observed temporal changes should be interpreted as institutional trends rather than patient-level treatment effects. Because both the intervention and the outcome were measured at the level of the calendar year rather than the individual patient, the analysis is subject to aggregation bias, so that the association observed at institutional level need not hold for individual patients. Moreover, no clinical, functional, safety or economic outcomes were analysed, and the results therefore cannot address whether the reduction in transfusion was accompanied by unchanged or improved patient outcomes. Finally, the case-mix of a national tertiary referral centre, which manages a disproportionate share of complex and polymorbid patients, may further limit the applicability of our absolute transfusion rates to other settings. Nevertheless, the study represents a large real-world analysis conducted over multiple years and including several thousand major orthopaedic procedures, thereby providing valuable insight into the clinical effectiveness of PBM implementation in routine practice. Importantly, our findings further support the concept that PBM should not be regarded as a single isolated intervention, but rather as a coordinated multidisciplinary strategy.

## Conclusion

Progressive implementation of a multidisciplinary PBM program was associated with a substantial and sustained reduction in transfusion rates in major orthopedic surgical procedures, observed consistently across total hip and knee arthroplasty, as well as major spine surgery. The combination of preoperative anemia optimization, increasingly restrictive, symptom-based transfusion thresholds, a single-unit transfusion policy, routine TXA administration, meticulous hemostasis, and modern surgical and anesthetic techniques accompanied a marked decrease in allogeneic blood utilization. PBM should therefore be considered a standard component of contemporary orthopedic perioperative care, and prospective patient-level studies including clinical, safety and economic outcomes are warranted to confirm and extend these observations.

## Data Availability

The data analyzed in this study is subject to the following licenses/restrictions: The data used and/or analyzed in the present study are available from the corresponding author on a reasonable request. Requests to access these datasets should be directed to matic.kolar@kclj.si.
